# *Tragopogon coelesyriacus Boiss.* attenuates ethanol-induced gastric ulcer *in vivo*: modulation of oxidative stress, inflammation, and apoptosis

**DOI:** 10.1371/journal.pone.0355381

**Published:** 2026-09-16

**Authors:** Hanan Ibrahim Alsharif, Mohammad Khaled Abu-Sini, Abdulrahman Ismael, Naz Farhad Abdulqadir, Gharieb S. El-Sayyad, Ahmed A.J. Jabbar, Sara Basil Abu Jalboush, Rawan Fahad Mosallam Almehmadi, Talal Salem Al-Qaisi, Khalid M. Alqaisi

**Affiliations:** 1 Department of Chemistry, College of Science, University of Jeddah, Jeddah, Saudi Arabia; 2 Department of Pharmacy, Faculty of Pharmacy, Al-Zaytoonah University of Jordan, Amman, Jordan; 3 Department of Community Health Nursing, Cihan University-Erbil, Erbil, Iraq; 4 Department of Medical Laboratory Technology, Erbil Technical Health and Medical College, Erbil Polytechnic University, Erbil, Iraq; 5 Department of Biology, College of Science, Imam Mohammad Ibn Saud Islamic University (IMSIU), Riyadh, Saudi Arabia; 6 Department of Medical and Clinical Laboratory Technology, Faculty of Allied Medical Sciences, Applied Science Private University, Amman, Jordan; 7 Department of Biomedical Sciences, College of Health Sciences, Abu Dhabi University, Abu Dhabi, United Arab Emirates; Noorda College of Osteopathic Medicine, UNITED STATES OF AMERICA

## Abstract

*Tragopogon coelesyriacus* is a traditional herbal medicine used for gastrointestinal disorders, mediated by its phytoconstituents. This study explores acute toxicity and gastroprotective potency of methanolic extracts of *Tragopogon coelesyriacus* (METC) in rats. Rats were aligned in 5 groups and pretreated with either normal saline, lansoprazole, or METC (200 and 400 mg/kg) before 5 mL/kg ethanol-induced gastric ulceration. The two-week toxicity study for METC (2 and 5 g/kg) showed no physiological alterations, behavioral changes, or morbidity in rats. Pretreatment with METC (200 and 400 mg/kg) improved gastric defense factors (mucus secretion and total stomach acidity), limiting mucosal penetrations and the lesioned areas. The histopathological results showed that METC (200 and 400 mg/kg) suppressed ethanol-induced gastric tissue alterations, indicated by increased gastric protective percentages (51.02% and 65.67%), less submucosal edema, epithelial exfoliation, lower **mucous metaplasia, and necrotic areas.** METC **supplementation (200 and 400 mg/kg) restored the level of** superoxide dismutase and catalase, while reducing the MDA by 29.84% and 48.64%, respectively. This was parallel with the anti-inflammatory effects of METC (200 and **400 mg/kg),** decreasing TNF-α by 34.59%, 47.36%; IL-6 by 24.95%, 30.32%, and increasing IL-10 by 73.94%, 179.92%, respectively, relative to ulcer controls. Additionally, METC (200 and 400 mg/kg) remarkably modulated apoptotic proteins, lowering Bax by 31.10% and 45.50%, and increasing HSP 70 by 28.12 and 65.5%, respectively. The outcomes highlight the significant gastroprotective potential of *Tragopogon coelesyriacus*, which was mainly associated with its antioxidant, anti-inflammatory, and anti-apoptosis properties, making it a viable source for gastric ulcer management.

## Introduction

Gastric ulcer remains the most prevalent gastrointestinal disorder with a nearly 10% incidence rate worldwide, which progresses into a lasting course of disease with increased incidence rates and numerous complications [1]. The disruption of a delicate balance between gastric defense barriers and aggressive factors triggers its pathogenesis. The mucosal defensive factors, such as mucus (mucopolysaccharides) and bicarbonate/prostaglandin secretion, endogenous antioxidants, epithelial regeneration/mucosal perfusion, maintain its gastric integrity. While risk factors such as *Helicobacter pylori* infection, NSAIDs, smoking, and excessive drinking promote stress factors, such as hypersecretion of stomach acid/pepsin, dysregulation of bile salts, ROS accumulation, and inflammation, they all participate in the ulcerogenesis. Alcohol overconsumption has been regarded as a primary exogenous inducer of gastric ulcer, as it can attenuate mucosal barriers by lowering mucus/bicarbonate production, provoke mucosal edema, enhance inflammatory cell infiltration, and impair microcirculation [2]. At the same time, stomach tissues are subsequently exposed to epithelial necrosis/apoptosis, free radical generation and oxidative stress, and severe inflammatory conditions. Moreover, ethanol-induced gastric ulcer is considered a reliable model for evaluating therapeutic/gastroprotective effects of a potential active ingredient due to its characteristic resemblance to human gastric ulcer [3].

The ethanol-induced gastric ulcer pathogenesis has been speculated by interrelated pathway alterations associated with oxidative stress, inflammation, as well as apoptotic-related pathways [4]. Ethanol is the widely used chemical to induce gastric ulcers in animals to study the anti-ulcer potential of the compound of interest. Ethanol can alter gastric defense barriers, cell membrane damage, dehydration, and induce cell cytotoxicity directly or indirectly via the generation of reactive oxygen species (ROS) that initiate oxidative stress and trigger inflammation [4]. The rapid mucosal barrier breakdown increases permeability to gastric acids and leukocytes (inflammatory cells) that promote degranulation of existing **mast cells** and the activation of **macrophages to release vasoactive products (leukotrienes,** TN**f-**α, **and** IL**-6 cytokines). These initiated cascade results in mucosal necrosis, erosion, and submucosal edema that ultimately induce gastric ulcers. Furthermore,** ethanol-induced gastropathy has been linked with its promoting apoptosis in the injury site by enhancing the overexpression of pro-apoptotic Bax (BCL2 Associated X) and suppression of cell survival HSP 70 (Heat shock protein 70) protein [5].

The current pharmaceuticals for gastric ulcers include H2-receptor antagonists (cimetidine and famotidine), antacids (e.g., aluminum/magnesium hydroxides), proton pump inhibitors (pantoprazole and lansoprazole), and mucosal protectives (sucralfate and bismuth subcitrate) [6]. However, frequent use of these stomach therapeutics has been associated with numerous side effects, including arrhythmias, hypersensitivity, impotence, hematological disorders, osteoporosis, kidney disorders, and gynecomastia. These after-effects of anti-ulcer drugs highlight the urgent need for pharmacologically better and safer therapeutics [7,8]. Natural products, particularly from herbal medicine, have gained renewed interest as promising candidates due to their increased pharmacodynamic potentials and favorable safety margins [9,10]. Despite the lack of a standard comprehensive pharmacovigilance of medicinal plants, global scientists are increasingly evaluating their therapeutic efficacy and possible adverse events [11,12].

*Tragopogon coelesyriacus* (Asteraceae family) is a pharmacotherapeutic herb with grass-like leaves, which were consumed as vegetables in Turkey and neighboring countries for many health purposes. In Turkish traditional therapies, some species of *Tragopogon* are ingested for anthelminthic purposes and to alleviate abdominal pain. Moreover, leaves of seven Tragopogon species have been served as fresh and an infusion to alleviate stomach and intestinal disorders [13]. *T. coelesyriacus* is also highlighted as one of the medicinal plants of ancient *materia medica* (a pharmacopeia of drugs written in the first century AD) [14]. Recent biological investigation unveiled numerous biological potentials of *Tragopogon species,* including antitumor, anti-inflammatory, antimicrobial, antihyperlipidemic, wound healing, enzyme inhibitor, and hepatoprotective actions, but studies on its possible toxicity effects are scarce [15]. Previous studies have determined numerous phytochemicals in *T. coelesyriacus*, including flavonoids and phenolics (chlorogenic acid, quinic acid, ferulic acid, luteolin, rosmarinic acid, and vitexin) [16,17]. However, the acute toxicity and gastroprotective effects of *T. coelesyriacus* have not yet been reported in an experimental gastric ulcer model.

Hence, this study was geared to explore the acute toxicity and gastroprotective effects of METC in ethanol-stimulated gastric ulcers. In addition, the association of gastroprotective efficacy with its antioxidant, anti-inflammatory, and anti-apoptosis action was investigated.

## Materials and methods

### Plant collection and extract

**Fig 1:**
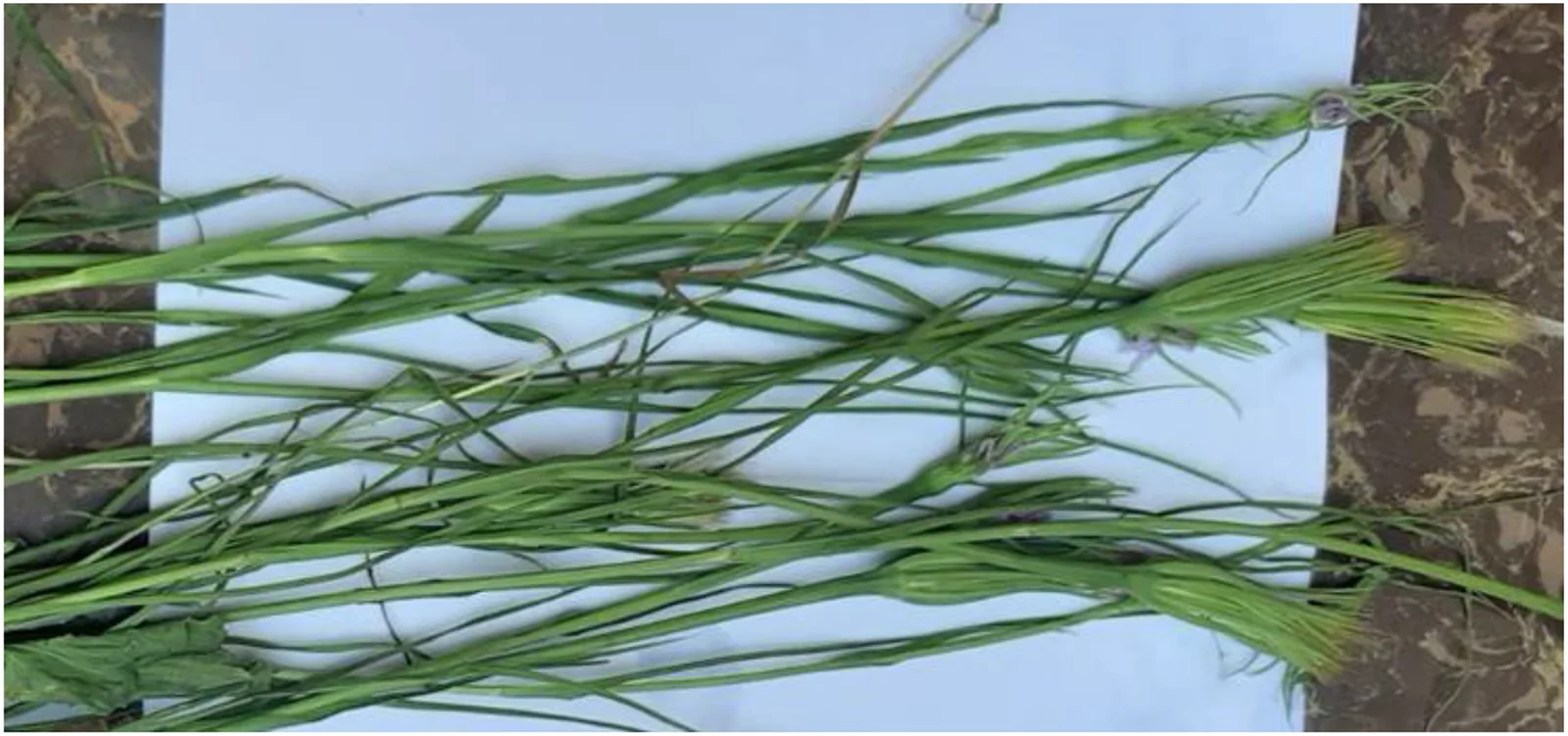
Aerial parts of *T. coelesyriacus* Boiss. was collected from Safeen Mountain, Erbil, Iraq (Photographs taken by A.A.J.).

The aerial parts of *T. coelesyriacus* (Fig 1) were collected from Safeen Mountain, Erbil, Iraq (altitude: 36.308398, latitude: 44.435329). The plant was authenticated by taxonomist Dr. Abdullah Sh. Sardar and the voucher number (594) were taken from the herbarium unit/Salahaddin University. The collected parts were washed, dried in the shade, and converted into a fine powder using a mill grinder. Methanol 95% was used as a solvent because of its higher polarity, which can dissolve a wide variety of phytochemicals, while the 5% water aids in plant cell penetration, and the solvent has a low boiling point (64.7° C) that facilitates evaporation [18]. An amount of 100 g of dried powder was mixed with methanol (95%) using a magnetic stirrer overnight (3x). The mixture solution was subjected to a gravity filtration procedure using Whatman paper (No. 4), and complete solvent dissociation was achieved using a vacuum at 40 °C [19]. The obtained crude extract (yield 18.5%) was sealed in dark vials (−20 °C) for later experiments. The doses of 2 and 5 g/kg for the acute toxicity test were selected according to OECD No. 423 guidelines [20], which were used along with the previous studies on *T. coelesyriacus* [15,16], to calculate employed doses, 200 or 400 mg/kg METC for the gastroprotective study.

### Chemicals and kits

The Methanol (>99%) has been purchased from Sigma Aldrich. The specialized ELISA rat kits for estimating antioxidant and relevant cytokines were purchased from Solar Biotech Co. (Beijing, China).

### Animals and ethics

Sprague Dawley adult rats (180–200 g; 7–9 weeks) were adopted in the research Centre/Erbil Polytechnic University. The animal use/care complied with ARRIVE 2.0 and the humane endpoint recommendations [21,22] and the study protocols were confirmed by the Ethics Committee of Erbil Polytechnic University (BIO, 67; 12/3/2025).

### Acute toxicity test

The present toxicity evaluation considered the applied standards recommended by OECD [23]. Two different doses (2 and 5 g/kg) were administered as a single dose to determine the (lethal dose 50%) LD_50_ level. Thirty-six male and female rats were randomly divided into 3 groups (n = 12 of both males and females in each group, separately caged, 6 rats/cage), fasted overnight, and then administered a single dose of either normal saline (group A), 2 g/kg METC (group B), or 5 g/kg METC (group C). After treatments, food was allowed for an additional 3 hours to allow complete absorption of the extracts. Rats were regularly observed (8 h) for any toxic signs, including abnormal behavioral (lethargy, head-burying, tremors, convulsions, weakness, loss of appetite, imbalance) and physical alterations (weight loss, bleeding, paralysis, paralysis, irregular breathing, salivation). Nonetheless, animals did not develop any pre-set physiological or behavioral limits that obligate us to reach a humane endpoint. At the end of the study, rats were injected with anesthesia prepared from 3 mg/kg xylazine and 30 mg/ kg ketamine to alleviate suffering [24], and they were subjected to euthanasia by decapitation using a sharp razor blade. The dissected liver and kidneys were processed for histopathological comparison using an H&E staining approach. In addition, blood samples were taken before euthanasia for some biochemical tests [25].

### Gastroprotective experiment

#### Experimental design.

Sprague Dawley rats (males weighing 180–200 g) were adopted in Erbil Polytechnic University, and they were housed in an animal unit with a suitable environmental condition (26–30 °C and 12 hr light/night cycle) in stainless steel wire cages. The two-week toxicity experiment determined an increased safe margin of METC (5 g/kg) in rats. In addition, previous studies have highlighted the increased therapeutic efficacy of METC in different inflammation-related disease models [16]. Therefore, the current study considers 200 and 400 mg/kg METC for evaluating its gastroprotective effects based on the current acute toxicity results and previous studies [15,16], 5mL/kg anhydrous ethanol as an inducer [26], and 30 mg/kg lansoprazole as the reference drug [5]. After the adaptation procedure, thirty male rats were randomly subjected to five groups (n = 6 each group/cage) and treated for 7 days as follows; control group (10% tween 20), ulcer control (10% tween 20), lansoprazole group (30 mg/kg lansoprazole), METC low dose (200 mg/kg METC), and METC high dose (400 mg/kg METC). After the final treatment, rats were fasted (24 h), and they received either saline (control) or absolute 5 mL/KG ethanol (the other four groups) as recommended [26]. One hour later, rats were injected with anesthesia prepared from 3 mg/kg xylazine and 30 mg/ kg ketamine to alleviate suffering [24], and they were humanely euthanized by decapitation using a sharp razor blade. The dissected stomachs and serum samples were obtained for relevant histopathological and biochemical analysis [27].

#### Gross and ulcer score study.

Stomachs were opened at the greater curvature, and the mucin/mucus content of each stomach was collected by scraping slides against the gastric linings, and the collected amount was estimated using an electric balance. Gross views of each stomach were photographed after opening at the greater curvature, flattened against ice packs, and Image-J software was employed to calculate the GU area.

The ulcer index of each stomach was determined employing an arbitrary scale, as explained elsewhere [28]. Briefly, 0 score for health stomach; score 0.5–1 for areas with hyperemia; score 1–2 for areas with hemorrhagic spots; score 2–3 for small ulcer areas; score 3–4 for several small ulcers; score 4–5 for 1–5 small and 1–3 large ulcers; score 5–6 for several large ulcers; score 6 for enlarged ulcers/perforations. The ulcer control scores were used against other treated rats to estimate the percentage of protective areas using Eq. (1)


$$\begin{array}{c} Protective\;\%=\frac{UI\;of\;vehicle-UI\;of\;group\;x}{UI\;of\;vehicle}\times \text{100} \end{array}$$ (1)

#### Evaluation of gastric contents.

The gastric contents were obtained from all rats, weighed, and subjected to centrifugation at 5000 rpm for 10 min. The separated supernatant was evaluated for pH (using a digital pH meter, Sartorius, Germany) and the total stomach acidity (TSA) using a previously detailed procedure [19]. Briefly, an aliquot of gastric juice (1 ml) was poured into a flask containing 1 ml of distilled water, followed by the addition of two drops of phenolphthalein and titrating them against 0.01 N NaOH to a permanent pink color. The consumed 0.01 N NaOH was recorded, and the total acidity was determined as mEq/L after some calculation using Eq. (2):


$$\begin{array}{c} TSA=\frac{\text{V}(\text{NaOH})\times \text{N}\times \text{100 mEq}/\text{L}}{\text{0}.\text{1}} \end{array}$$ (2)

Furthermore, the gastric contents were tested for the Alcian blue binding (ABB) capacity by applying the previous protocols [29]. Briefly, the glandular portion was mixed with Alcian blue mixture (10 mL of 0.1% w/v, pH 5.8) for 2 h. After removal of excess dye using sucrose solution (10 mL, 0.25 M), 10 mL of 0.5 M magnesium chloride was added and mixed for 30 min. After that, the mixture was poured into a flask containing 4 mL of diethyl ether on an electric shaker, incubated for 2 min, centrifuged at 4000 rpm for 10 min, and the absorbance was obtained at 580 nm. The ABB capacity was calculated, and the results were expressed as mg/gram of tissues.

### Histological analysis

The dissected stomachs were washed with saline and fixed in 10% formalin for 2 days. The tissue was sectioned and placed in plastic cassettes for the automated tissue processing using (Sakura, Japan). After that, tissue cassettes were paraffinized accordingly, and small sections (5 μm) were fixed on slides for staining with hematoxylin, eosin, and Periodic Schif stain. The slides were dried overnight in an oven, and they were screened by a blinded examiner for histopathological changes (three fields/animal). Histopathological scoring of tissue damage, including sloughing of the gastric epithelium, leucocyte infiltration, submucosal edema/penetration, congestion of gastric vessels, and gastric hemorrhage, was scored as follows: 0 = no change; 1= < 10–20% tissue damage; 2 = 21–30% tissue damage; 3 = 31–40% tissue damage; 4= > 40% tissue damage. For example, score 4 represents the highest epithelial desquamation of 2/3 lower lamina propria, and 0 represents intact epithelium [30]. Moreover, the amount of magenta color/PAS expression is an indication of mucopolysaccharide contents [28].

### Immunohistochemical analysis

Tissue pieces were immersed in paraffin, deparaffinized using xylene, followed by hydration using an ethanol series. The immunohistochemical procedure followed the protocols provided by the manufacturer. In brief, deparaffinized gastric tissues were blocked with 5% bovine serum albumin (BSA) for 2 h in Tris-buffered saline (10 mM) (pH 9.0) for 15 minutes to retrieve the antigens. After incubation of slides were cooled down to 37°C, and sections were blocked with endogenous peroxidase, incubated in 3% H2 O2 for 10 minutes at 37°C. After washing (2x) the slides with PBS, incubated at 4 °C overnight with either anti-Bax (1:500, Cat. No. K008076P) or anti-HSP70 (1:500, Cat. No. K012232RR) monoclonal antibody (Solar biotechnology, Beijing, China) [31]. After washing the slides, incubation with biotinylated goat anti-rabbit secondary antibody, and a second incubation with horseradish-peroxidase conjugated streptavidin solution for 10–15 min at 37 C. Accordingly, the slides were washed again with TBS, fixed via 0.02% diaminobenzidine (DAB) with 0.01% H_2_O_2_ for 10 min., counterstained with hematoxylin, and dried sections were mounted with DPX. The slides were screened by a blinded examiner under a Trinocular Zeedo microscope. For quantification, the immunohistochemical expression of immunopositive cells (brown colored granules) was estimated as the optical density of Bax and HSP-70 staining from seven fields/stomach using computerized Image-J software 1.54k (Bharti Airtel Ltd, New Delhi, India) [32].

### ELISA assays

The gastric tissue portions were washed and homogenized with **0.1M phosphate-buffered saline at 1:10 w/v, pH 7.4**, at 4 °C, centrifuged at 4500 rpm (15 min, 4 °C) [33]. The supernatant was examined for the content of SOD, CAT, MDA, as well as inflammatory cytokines TNF-α, IL-6, and IL-10, applying protocols available on commercial ELISA kits (Solar Bio Technology Co., Beijing, China) as mentioned elsewhere [34].

### Statistics

The laboratory results were handled by One-way analysis of variance (ANOVA) and Tukey’s HSD tests for comparison of group means after confirming certain assumptions (normality, Homogeneity of Variances, and independence). The designed graphs were possible via GraphPad Prism 9.1 and Bio Render. Values shown as mean ± SEM, and different significance levels were indicated in Table footnotes and legends as asterisks. *, p<0.05; **, p<0.01; ***, P<0.001; ****, p<0.0001.

## Results

### Acute toxicity

**Fig 2:**
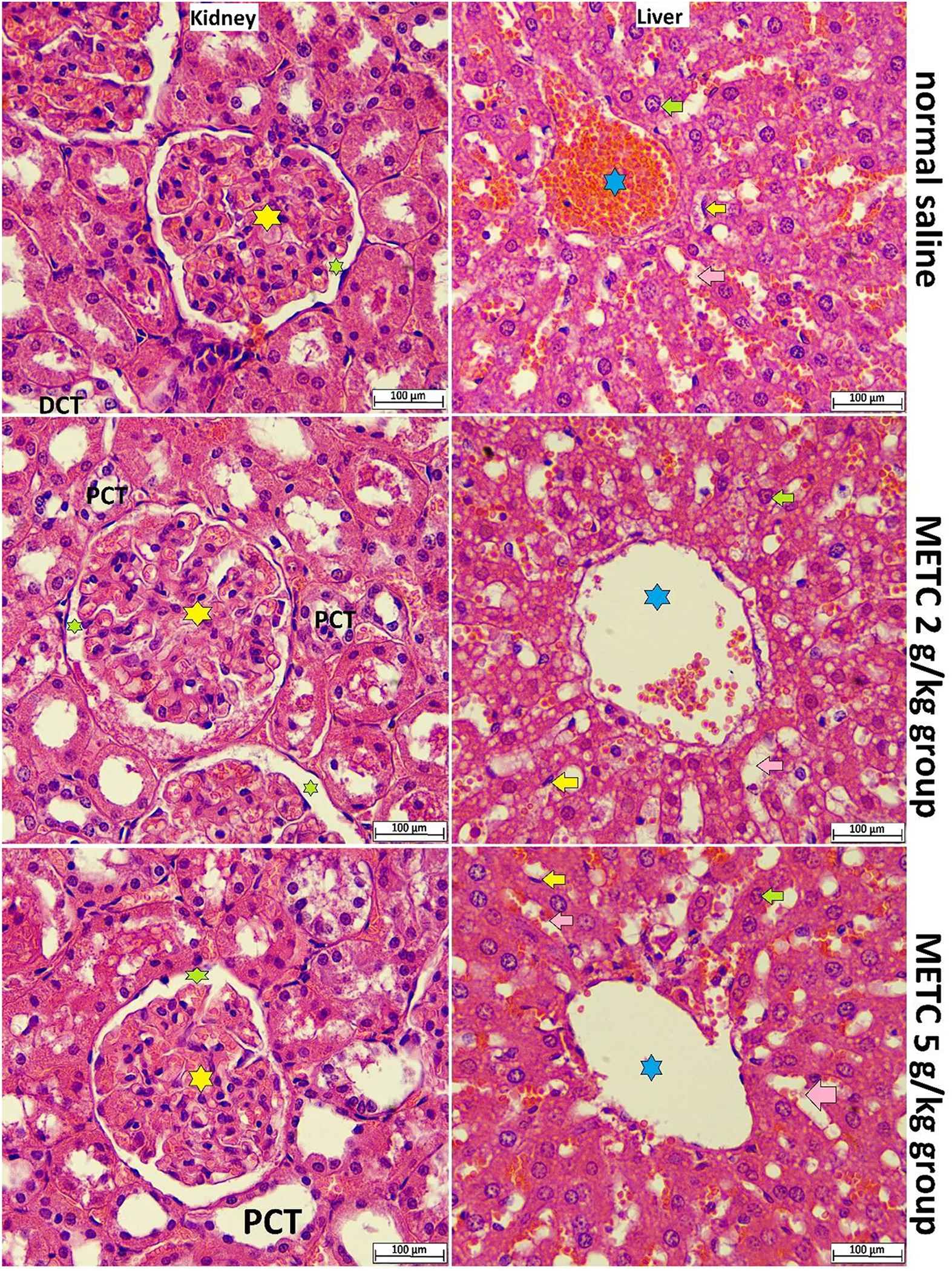
Microscopic views of the liver and kidney of rats (n = 12). blue star, central vein; green arrow, sheets of hepatocyte; yellow arrow, Kupffer cells; yellow star, glomerulus; pink arrow, sinusoids; DCT, PCT, distal and proximal convoluted tubules (H & E staining, 40x). There was no noticeable difference in liver and kidney tissue alignment between normal control and METC-treated rats.

All experimental rats ingested 2 and 5 g/kg of METC or normal saline and survived the 14 days of screening period. Indeed, drug-associated toxic signs such as stereotypic behavior, excitation, Straub tail, jumping, loss of traction, sedation, reduced muscle tone, tremors, defecation, salivation, and convulsion were not detected in supplemented and normal saline-treated rats. The histopathological screening of the liver and kidneys from METC or normal control did not find any altered tissue layers or changes, such as necrosis, lesions/hemorrhagic areas (Fig 2). The serum biochemicals (liver and kidney functional parameters) were found to be non-significantly (p > 0.05) different between normal control and METC-treated rats (S7, supplementary Table 1). The results provide a scientific ground for the safe dosage utilization of METC, with the lethal dose (LD_50_) being higher than 5 g/kg in rats.

### Assessment of gastroprotective activity

#### Gross evaluation.

**Fig 3:**
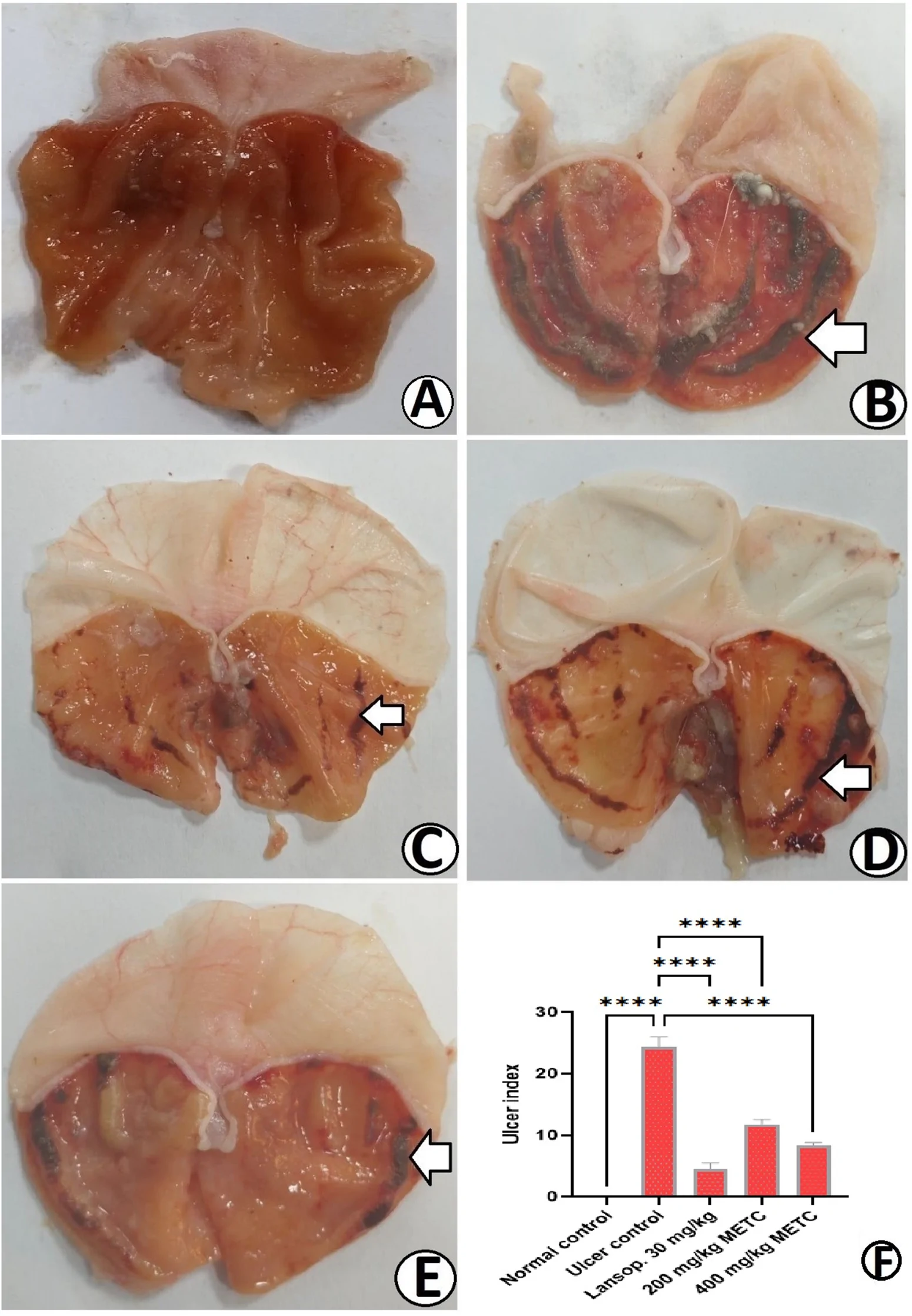
Stomach Gross views of different experimental rats used in the gastroprotective trial and quantitative analysis of ulcer index of experimental groups (A-F). A, normal control received 10% tween 20 + saline; B, ulcer control had 10% tween 20 + ethanol; C, lansoprazole group rats pretreated with 30 mg/kg lansoprazole+ ethanol; D, low dose METC received 200 mg/kg METC + ethanol (D); E, high dose METC had 400 mg/kg METC + ethanol. Ulcer controls experienced severe gastric tissue injury, denoted with numerous pinpoint red points, hemorrhage (bleeding), erythema (redness), and erosions (shallow breaks on the surface mucosa). These gastric tissue alterations were attenuated as a result of lansoprazole or METC pretreatments in a dose-related fashion. Panel F shows that METC-treated groups (200 and 400 mg/kg) had significantly lower ulcer index (11.9 and 8.34) compared to that (24.3) of ulcer controls (vehicle). Values shown as Mean ± SEM (n = 6). *, p<0.05; **, p<0.01; ***, P<0.001; ****, p<0.0001. (Photographs taken by A.A.J.).

The dissected stomachs are seen with different levels of hemorrhagic/lesion areas due to various pre-treatments and ethanol delivery. Ulcer control rats exhibited severe mucosal hemorrhage areas presented as an extended thick line across the gastric axis, deeper sores, bleeding, folded mucosa, and shallow breaks in the lining of the gastric layers. As expected, the gastric lining of normal controls was seen as pink, healthy mucosa with usual gastric folds. Pretreated rats with either lansoprazole or METC exhibited mild-moderate gastric tissue injury, indicated by more gastric folds, fewer hemorrhagic/lesion areas, more flattened areas, fewer swollen/pinpoint red spots, and significantly lower ulcer index than ulcer controls (Fig 3A–3F). Rats pretreated with 400 mg/kg METC had comparable gastric mucosal injury to lansoprazole-treated rats, shown by reduced gastric lesions and lack of mucosal bleeding, and comparable healthy/pink gastric mucosa.

### Effect of METC on the gastric contents

The mucus weight, gastric pH, gastric injury area, and inhibition percentage are shown in Table 1. Normal control rats exhibited values within standard ranges regarding mucus weight, gastric pH, TSA, and ABB, which had the highest gastric pH, mucus weight, and the lowest total stomach acidity compared to the other treated rats. The ulcer control had significantly (p < 0.05) lower mucus weight, less ABB by 42.46% lower, reduced gastric pH, while having higher TSA by 94.36%, compared to normal controls. In contrast, lansoprazole or METC (200 and 400 mg/kg) pretreatment reduced TSA by 40.61, 24.85, 29.79%, respectively, and up-regulated ABB capacity by 28.92, 15.61, 22.01%, respectively, compared with ulcer control values as positive standards. Moreover, ulcer control rats showed increased gastric tissue injury with an estimated value of 24.3 mm^2^; however, lansoprazole and METC pretreatments (200 and 400 mg/kg) inhibited the ulcer index by 81.46, 51.02, and 65.67%, respectively.

**Table 1:** Effect of METC on gastric measurements.

Groups	Mucus weight (g)	Gastric juice pH	Ulcer index	Protective %	TSA (mEq/L/100 g)	mg Alcian blue capacity (ABB)/ g tissues
**Normal saline group**	0.356 ± 0.05^a^	3.8 ± 0.59^a^	–	–	63.54 ± 3.2^a^	483.2 ± 4.2^a^
**Ulcer control**	0.055 ± 0.003^a^	1.54 ± 0.54^b^	24.3 ± 2.43^a^	–	123.5 ± 4.1^d^	278.0 ± 4.7^e^
**Lansoprazole+ ethanol**	0.288 ± 0.05^b^	2.40 ± 0.50^a^	4.5 ± 1.2^b^	81.48^a^	73.34 ± 5.4^b^	358.4 ± 4.3^b^
**200 mg/kg METC + ethanol**	0.178 ± 0.03^b^	1.82 ± 0.43^d^	11.9 ± 1.1^c^	51.02^b^	92.81 ± 4.2^c^	323.9 ± 4.8^d^
**400 mg/kg METC + ethanol**	0.231 ± 0.04^b^	2.12 ± 0.33^a^	8.34 ± 2.4^d^	65.67 ^c^	86.70 ± 5.4^c^	339.2 ± 5.9^c^

Values presented as mean ± SEM. Groups sharing the same superscript within a column are not significantly different, *p* > 0.05. TSA, total stomach acidity; ABB, Alcian blue binding capacity, shows the quantity of protective **gastric wall mucus**, where higher values/binding capacity mean a thicker, healthier mucosal layer.

### Microscopic tissue alteration and mucus/glycoprotein content

**Fig 4:**
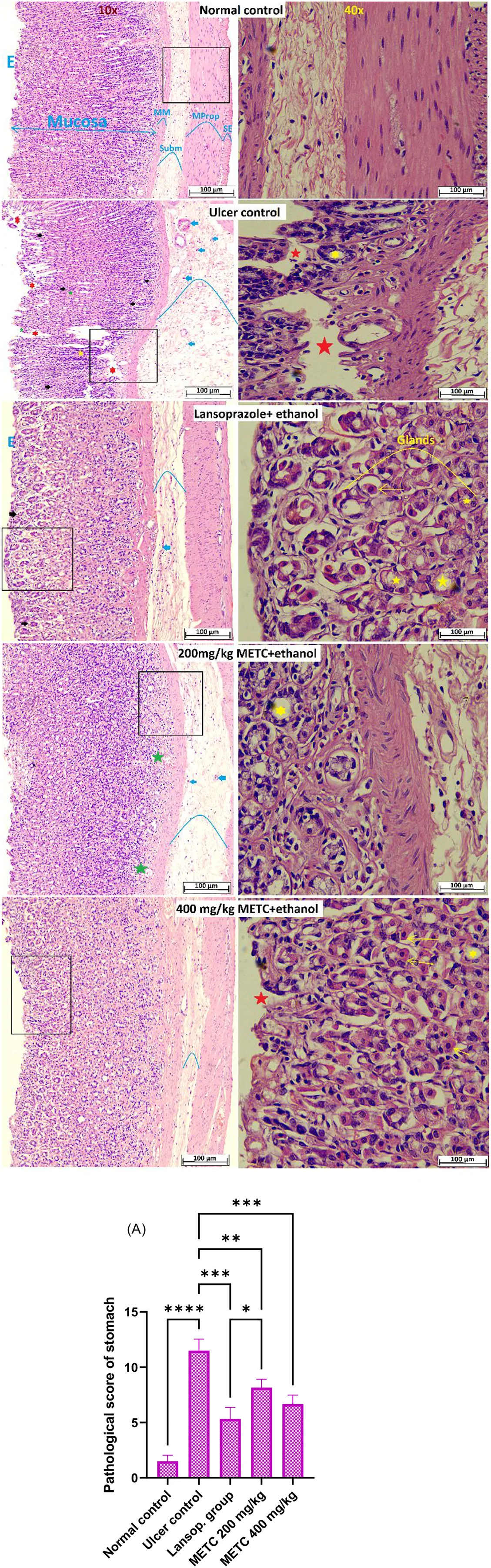
H&E-stained photomicrographs (10x and 40x) of stomach tissues obtained from different experimental rats. Normal control exhibited a full thickness of mucosa with normal gastric tissue layers. Ulcer control showed increased epithelial disruption/erosion and exfoliation of necrotic cells (red star) as well as increased inflammatory cell infiltration (blue arrow) in oedemic submucosal (blue curve), congested blood vessel congestion (green star), and apoptotic bodies (black arrow). The lansoprazole (c), METC (200 and 400 mg/kg)-treated groups show less submucosal edema, fewer inflammatory cells, fewer vacuolated parietal cells (yellow arrow), and more intact epithelial (E) layers. Subm, sub-mucosa; MM, muscularis mucosa; Mprop, muscularis propria; SE, serosa; yellow star, chief cells. (A), shows pathological score for gastric damage, including sloughing of the gastric epithelium, leucocyte infiltration, submucosal edema/penetration, congestion of gastric vessels, and gastric hemorrhage, was scored as follows: 0 = no change; 1= < 10-20% tissue damage; 2 = 21-30% tissue damage; 3 = 31-40% tissue damage; 4= > 40% tissue damage. Values shown as Mean ± SEM (n = 6). *, p<0.05; **, p<0.01; ***, p<0.001; ****, p<0.0001.

Applying H&E- and PAS-stained procedure, microscope screening of gastric sections unveiled that normal control rats exhibited normal gastric tissue histological features; the gastric mucosa constitutes from epithelium, muscularis layer, and lamina propria. The gastric glands are positioned beneath the upper layer of the lamina propria and are accessed via short/thin pits into the lumen. The outer layer that lines the epithelium is occupied by mucus simple/columnar cells that are characterized by foamy cytoplasm/oval nuclei. Gastric glands are observed with an inner isthmus and surface mucous cells, followed by the middle neck (containing mucous neck and parietal cells), and the outer basal area lined by parietal/chief cells. Parietal cells are recognized with central nuclei and acidophilic cytoplasm, unlike chief cells, which are tiny cells with basal nuclei and basophilic cytoplasm. Histopathological screening of the ulcer control group evidenced substantial alteration, severe erosion, and irregularities in the cells that caused architecture alteration of the mucosal layer, including the spread of desquamated cells in the muscularis mucosa; enlarged, extravasation of RBCs in congested blood vessels; pyknotic surface epithelial cells; numerous inflammatory cell infiltrations in the lamina propria, areas of tissue loss, and vacuolated gastric glands. In contrast, rats receiving lansoprazole or METC (200 and 400 mg/kg) revealed moderate-mild degenerated cells, including vacuolated parietal cells, and a few mononuclear cell invasions, and fewer extravasated RBCs of congested blood vessels seen in the basal part. Moreover, the stomach tissues of METC-treated rats had more intact mucosal areas with regularly arranged fundic glands (Fig 4).

**Fig 5:**
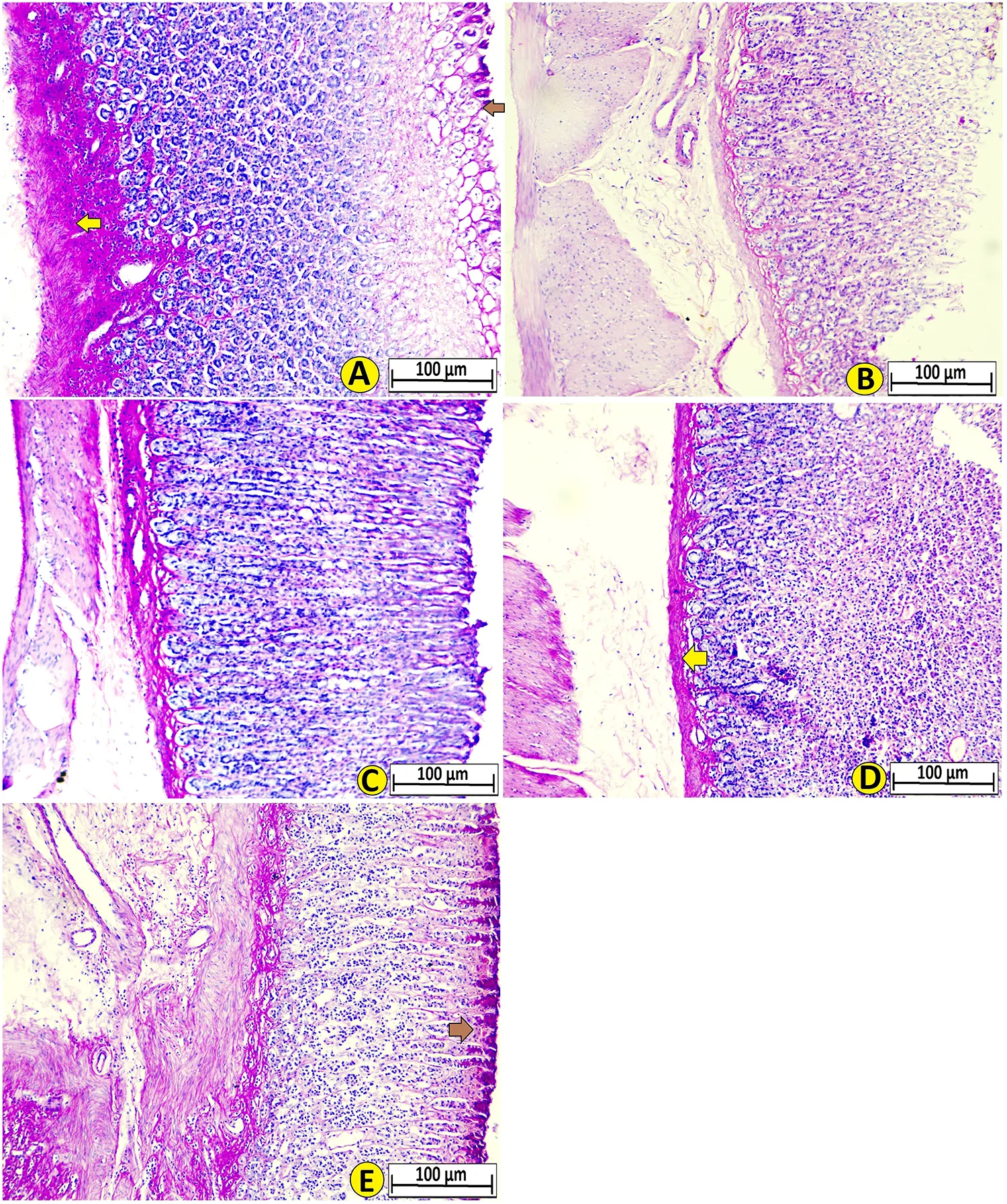
Gastric tissue expresses different levels of PAS stains. A, normal control received 10% tween 20 + saline; B, ulcer control had 10% tween 20 + ethanol; C, lansoprazole group rats pretreated with 30 mg/kg lansoprazole+ ethanol; D, low dose METC received 200 mg/kg METC + absolute ethanol (D); E, high dose METC had 400 mg/kg METC + absolute ethanol. Ulcer control group unveiled a decreased amount of PAS stains in their gastric mucosa. METC pretreatment enhanced gastric glands and increased mucin production (intense magenta color).

The histopathological screening employed the PAS stain procedure to observe changes in the mucin depletion in the inner mucosal membrane. As indicated in Fig 5, the area of purplish red on the gastric tissues dissected from ulcer controls was notably lower than the amount detected for normal controls. Nevertheless, lansoprazole and METC pretreatment restored the depleted potentials of mucin production from the gastric gland/mucoid cells. In particular, after intragastric delivery of 400 mg/kg METC, the positive rats of PAS expression were significantly modulated, which were very comparable to that of the normal control group.

### Effect of METC on immunostaining

**Fig 6:**
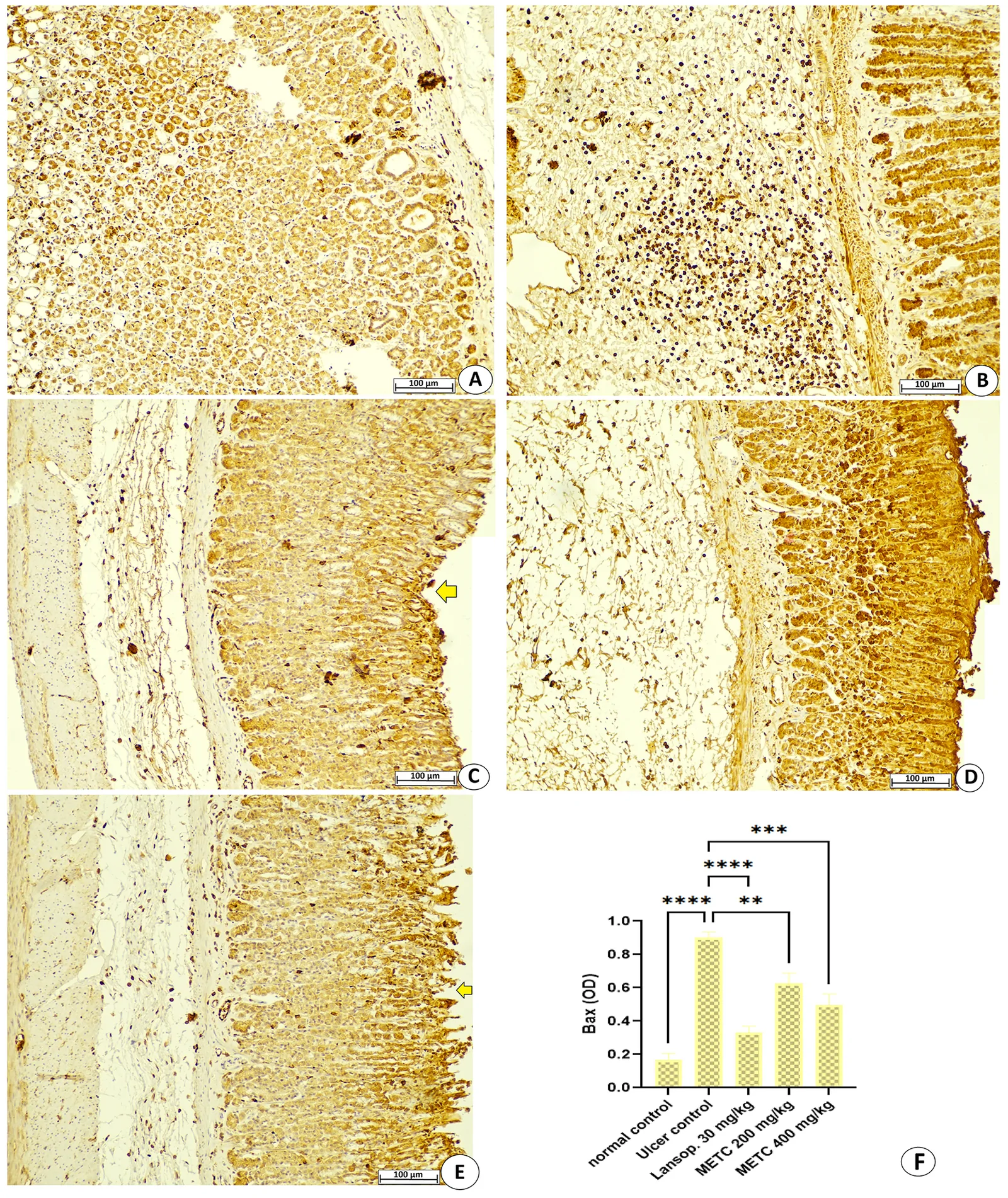
Effect of METC on apoptotic Bax expression in gastric tissues of experimental rats (n = 6). A, normal control received 10% tween 20 + saline; B, ulcer control had 10% tween 20 + ethanol; C, lansoprazole group rats pretreated with 30 mg/kg lansoprazole+ ethanol; D, low dose METC received 200 mg/kg METC + absolute ethanol (D); E, high dose METC had 400 mg/kg METC + absolute ethanol. The ulcer controls showed a significantly higher number of brown-stained cells in their tissues compared to healthy controls. METC pretreatment showed resistance to ethanol-mediated Bax protein alterations, indicated by significantly less optical density of Bax-represented cells than ulcer controls in a dose-related manner (F). (n = 6). *, p<0.05; **, p<0.01; ***, p<0.001; ****, p<0.0001.

**Fig 7:**
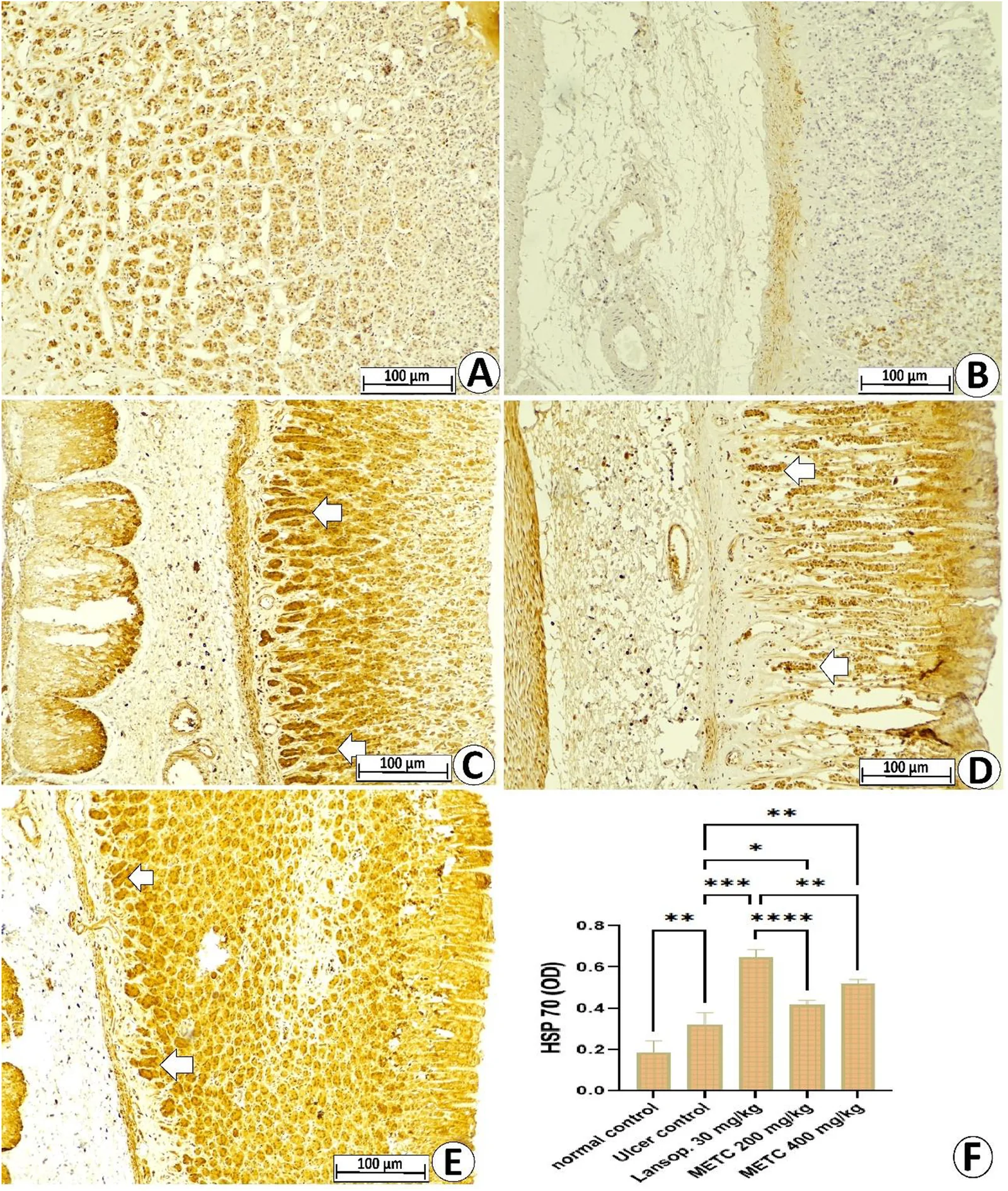
Immunohistochemical representation of HSP 70 proteins in gastric tissues of experimental rats (n = 6). A, normal control received 10% tween 20 + saline; B, ulcer control had 10% tween 20 + ethanol; C, lansoprazole group rats pretreated with 30 mg/kg lansoprazole+ ethanol; D, low dose METC received 200 mg/kg METC + absolute ethanol (D); E, high dose METC had 400 mg/kg METC + absolute ethanol. The ulcer controls exhibited reduced expression of HSP 70 proteins. While pretreatment with lansoprazole or METC (200 and 400 mg/kg) increased HSP 70 expression, shown by significantly (P < 0.05) higher optical density than that of ulcer controls (A-F). *, p<0.05; **, p<0.01; ***, p<0.001; ****, p<0.0001.

The Bax proteins in the healthy group’s gastric tissues were found as mild brown staining areas in the cytoplasm. Notably, gastric tissues from ulcer controls showed increased optical density of Bax proteins (increased by 442.16% compared to the healthy group) with numerous brown-stained cytoplasmic areas. By comparison, lansoprazole or METC pretreatment (200 mg/kg and 400 mg/kg) relatively reduced Bax protein expression by 63.30%, 31.10%, 45.50%, respectively, compared to ulcer controls (Fig 6). In addition, the HSP 70 protein expression in ulcer controls was significantly (P < 0.01) lower by 72.97% compared to the healthy group, while lansoprazole, METC 200, and 400 mg/kg increased expression of HSP 70 proteins by 103.12%, 28.12%, 65.5%, respectively, compared to ulcer control. The data clearly highlight the modulatory effects of METC on apoptotic proteins in a dose-related fashion (Fig 7).

### Effect of METC on oxidative stress

**Fig 8:**
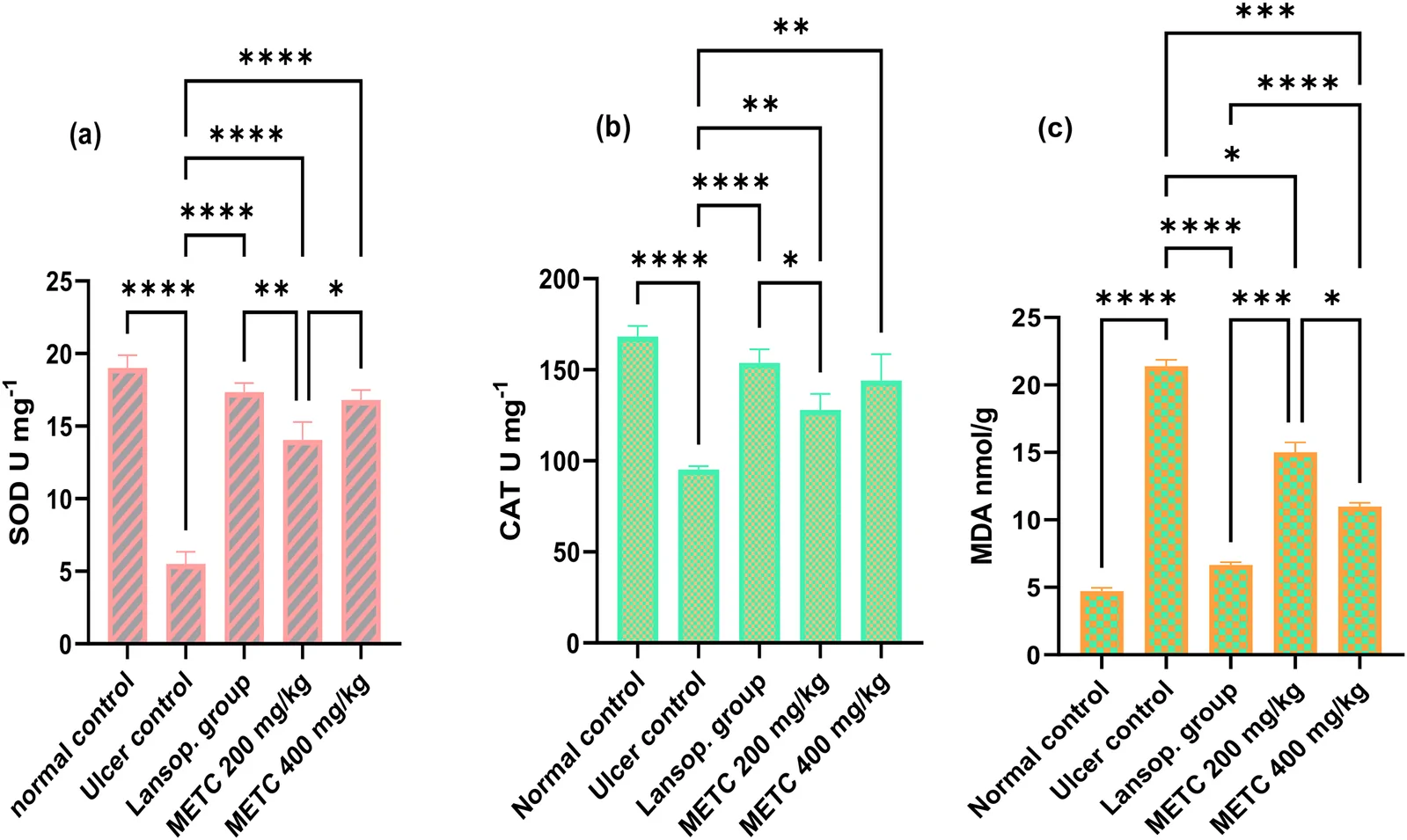
Effect of METC on gastric tissue oxidative stress marker and antioxidants: (a) SOD activity, (b) CAT activity, and (c) MDA levels. Pretreatment with METC enhanced antioxidant expression that reduced oxidative-induced tissue injury, indicated by significantly higher SOD, CAT, and lower MDA levels than those of ulcer controls. Values shown as mean ± SEM (n = 6/group) and the significance levels presented as. *, p<0.05; **, p<0.01; ***, p<0.001; ***, p<0.0001.

The GU-provoked by ethanol was associated with increased oxidative stress, denoted by reduced endogenous antioxidants (lowered SOD 71.05% by and CAT by 43.40) and significantly (*p* < 0.0001) increased MDA (increased by 353.9%) contents in ulcer controls compared to normal controls. Such oxidative stress-mediated tissue damage was attenuated by pretreatment of lansoprazole or METC (200 and 400 mg/kg), up-regulating SOD by 215.45%, 155.39%, 205.40%; CAT by 61.48, 34.50%, 151.39%; and reducing MDA contents by 68.90, 29.84, 48.64%, respectively, compared to the ulcer control (Fig 8).

### Effect of METC on inflammation

**Fig 9:**
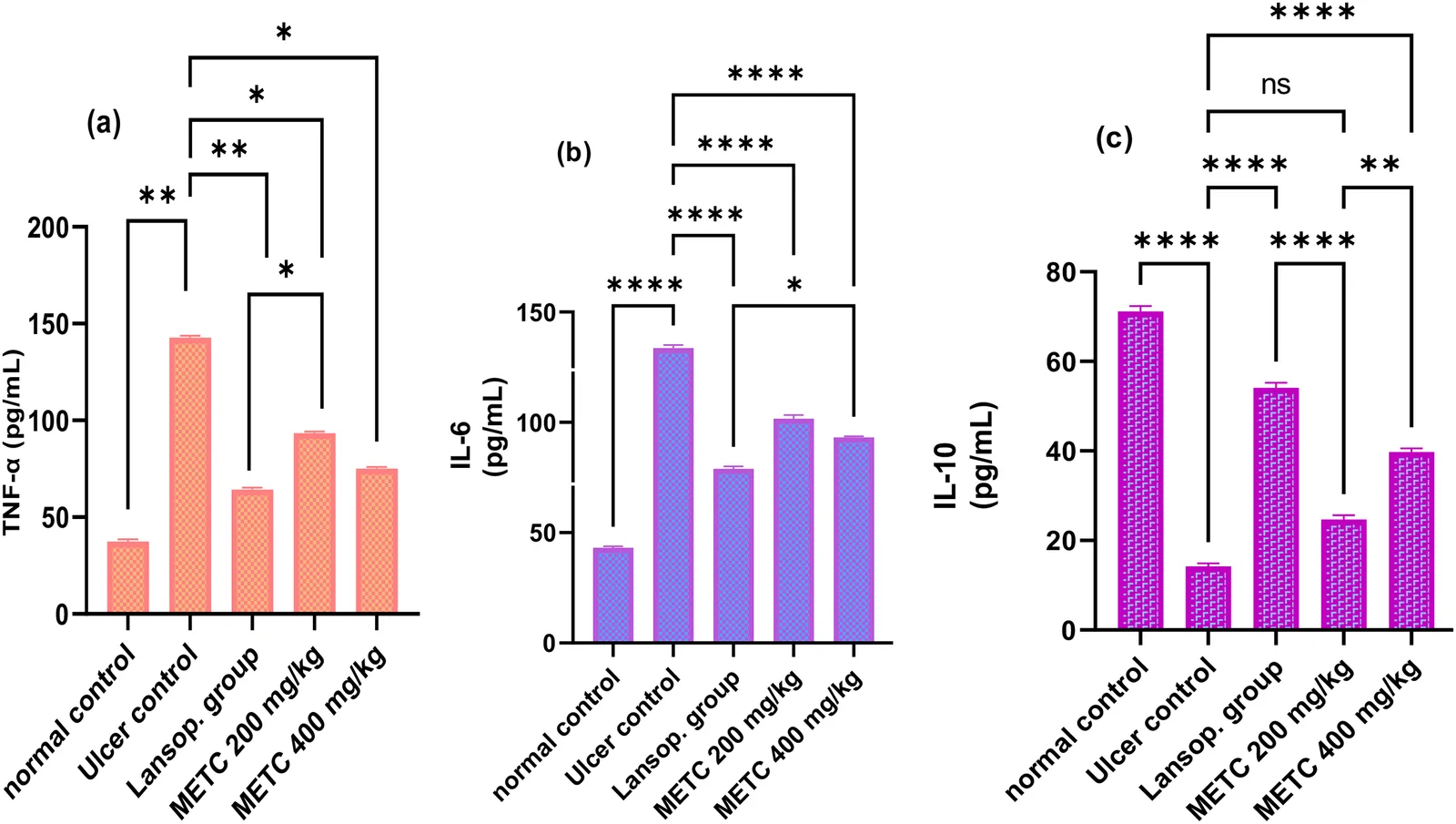
Effect of METC on inflammatory cytokines in gastric tissue homogenates of rats (n = 6/group). (a) TNF-α concentration, (b) IL-6 concentration, and (c) IL-10 concentration. Pretreatment with METC attenuated EtOH-induced inflammation, denoted by a markedly lower TNF-α, IL-6, and significantly (P < 0.0001) higher IL-10 cytokines. Values shown as mean ± SEM (n = 6/group) and the significance levels presented as. *, p<0.05; **, p<0.01; ***, p<0.001; ****, p<0.0001.

The ethanol-induced GU model/ulcer control exhibited a severe inflammatory condition, shown by statistically increased pro-inflammatory cytokines (TNF-α by 281.63%, P < 0.01; IL-6 by 209.84%, P < 0.0001) and reduced IL-10 levels (80.04%, P < 0.0001), compared to normal controls. Rats pretreated with lansoprazole or METC (200 and 400 mg/kg) decreased the level of TNF-α by 54.94%, 34.59%, 47.36%; IL-6 by 40.96, 24.95%, 30.32%, respectively, compared to the ulcer control group. Moreover, lansoprazole or METC (400 mg/kg) pretreatment has increased IL-10 by 280.70%, 179.92%, respectively, compared to ulcer controls. While 200 mg/kg METC pretreatment non-significantly increased IL-10 by 73.94% compared to the value of ulcer controls. The data analysis presents substantial anti-inflammatory potentials of METC in a dose-related manner (Fig 9).

## Discussion

Herbal medicine has been employed as a therapeutic agent for managing different health disorders worldwide. According to the WHO, nearly 80% of the world’s population uses medicinal plants as their main source of curatives, especially those living in rural areas [35]. The lack of literature data regarding its safety dose, possible toxic effects, and biological potential on *T. coelesyriacus* led this investigation to inventory the herbal medicine used by folkloric practitioners to treat stomach aches and intestinal disorders, and evaluate its gastroprotective potential through a histopathological and immunohistochemical approach. Toxicity evaluation is considered a preclinical review that requires less time and is more cost-effective than clinical trials [36]. The intragastric delivery of a single dose of *T. coelesyriacus* extracts (2 and 5 g/kg) to rats did not provoke any observable toxicity signs either during the first 8 h of ingestion or during the 14 days of the trial. All rats were completely healthy at the end of the experiment, indicating no toxicity at a level of 5000 mg/kg, and the lethal dose (LD_50_) of the methanolic extracts is greater than 5000 mg/kg. The current results coincide with the previous data of Mojarrab et al., where no biochemical/organ changes or death occurred in rats ingested with 2500 mg/kg *Tragopogon* extract (*T. buphthalmoides*), and they expected that the LD50 would be higher than the tested dose [37]. Accordingly, rats injected with 250, 500, 1000, and 2000 mg/kg *Tragopogon Graminifolius* extracts did not display any toxic signs or abnormal behaviors, with zero mortality, even after the trial [38]. Applying the OECD’s Globally Harmonized System for chemical safety classification, *T. coelesyriacus* is considered practically non-toxic and is categorized as level 5 [23].

The *T. coelesyriacus* doses used in the gastroprotective study were based on the current acute toxicity results and the previous studies on Tragopogon extracts to ensure *in vivo* efficiency and safety [16,38]. Moreover, the dosages used in the current investigation can also be adjusted according to established human equivalent dose conversion methods to translate our preclinical results to potential human applications.

The gastric ulcers induced by absolute ethanol in rats are considered to be similar to the pathways that occur in human gastric ulcers, with comparable features of healing and recurrence [39]. In this gastroprotective trial, intragastric ethanol delivery provoked significant gastric tissue injury, indicated by several gross indications such as surface dark/red lesions, mucosal erosion, and flattened surface lining/reduced folds. While microscopic screening evidenced epithelial disruption, leukocyte infiltration, cellular exfoliation, and mucosal/submucosal edema [39]. The pharmacological treatment for gastric ulcers includes chemical synthetics that promote mucosal protection or reduce stress factors. Therefore, in this study, lansoprazole was used as a positive drug control, which decreases gastric acid secretion by creating a mechanical barrier and restores the gastric pH altered by absolute ethanol [40]. A large body of literature data supports medicinal plants as gastroprotective and curatives for gastric ulcers through improving the gastric defense system and regulating the production of mucopolysaccharides that are essential for balanced gastric pH [41]. Interestingly, METC pretreatment (200 and 400 mg/kg) attenuated ethanol-mucosal injury, shown by fewer observable gross lesions, lower ulcer index, and less structural tissue alterations (more healthy epithelial tissues in the upper portion of fundic glands and higher PAS/glycoprotein expression) compared to ulcer control rats. The present gastroprotective potential of *T. coelesyriacus* could be attributed to its phenolic contents (mainly, chlorogenic acid, quinic acid, and vitexin), which were regarded as anti-ulcer compounds in different gastric ulcer animal models [16,42,43]. Similarly, daily oral administration of *Tragopogon graminifolius* extracts 50, 100, and 150 mg/kg ameliorated ethanol-mediated gastric ulcers, which were mainly linked with its phytochemical potential in strengthening gastric defense barriers and regulating acid/mucus secretions [38]. Accordingly, *Tragopogon graminifolius oral treatments* (20, 30, or 50 mg/kg/d) attenuated 2,4,6-trinitrobenzenesulfonic acid-induced ulcerative colitis, which was explained by its increasing phenolic contents (560.67  ±  18.85 mg/g GAE) exhibiting significant antioxidant and anti-inflammatory potential capable of modulating nuclear factor-kappa B (NF-κB) and its related pathways [44].

The Apoptotic actions can take place as a normal physiological process in all gastric regions, predominantly in superficial gastric parts/glands, at a rate of 2−3% for all gastric cells [45]. However, this rate rapidly increases once the gastric mucosa is exposed to stress factors such as ethanol, causing exacerbation of mucosal injury or a delay in healing of existing injuries. Pro-apoptotic proteins such as Bax have been key players in intrinsic/mitochondrial apoptosis through enhancing the release of cytochrome c and activation of caspase-9 and other effector caspases such as caspase-3 [46]. Thus, apoptosis induction of gastric mucosal cells is considered a crucial factor associated with the initiation of gastric ulcers. Therefore, the prevention of premature mucosal cell apoptosis is considered another means of gastric ulcer inhibition. Previous data confirmed that up-regulation of Bax proteins enhances the gastric ulcer exacerbation, which was also the case in our ulcer control [47]. Moreover, HSP 70 protein is abundantly present in mammalian cells, acting as a cytoprotective agent, resisting oxidative stress damage, and preserving the structural integrity of normal cells as well as maintaining cellular proteostasis by aiding in protein refolding/removing damaged proteins, events which usually take place once the gastric mucosa is exposed to irritants such as ethanol or other stress factors. HSP 70 play important role in the gastric ulcer recovery by increasing the expression of PGE2 and enhancing other gastric defense mechanisms [48]. During ethanol-induced oxidative gastric tissue injury, ROS molecules are generated in tremendous amounts in ulcer areas, disrupting a delicate redox state as it surpasses the oxidation resistance of endogenous antioxidants; it impedes the expression of HSP 70 and promotes activation of Bax protein, making gastric mucosa more vulnerable to ulcerative damage [32]. In alignment with the above knowledge, the present ethanol delivery caused a notorious promotion of apoptotic-mediated tissue damage, indicated by up-regulated Bax and down-regulated HSP 70 protein expressions. Interestingly, METC treatment enhances a substantial reduction in apoptotic rate in gastric tissues, supported by a restoration of HSP 70 proteins and a substantial reduction of Bax expression, which can be attributed to the previously reported phenolic contents (mainly, chlorogenic acid, quinic acid, and vitexin) [49–51]. Accordingly, Xie et al. have shown significant modulatory potentials of *Tragopogon collinus* extracts on apoptotic proteins, including caspase-9/-3 activity, Bax and caspase-9/-3, Bcl-2, as well as regulation of other apoptosis-associated genes in mitochondrial mechanisms [52]. Similar effects of rich phenolic/flavonoid extracts of other plant species were reported in the ethanol-mediated gastric ulceration model [53].

Ethanol is metabolized throughout the gastrointestinal tract, mainly by the stomach, where it is absorbed and metabolized by gastric alcohol dehydrogenase (ADH), generating toxic compounds such as acetaldehyde that can provoke inflammation-related pathways. Ethanol metabolism also produces numerous ROS molecules (superoxide radicals) and stimulates lipid peroxidation (malondialdehyde) [54]. In this study, the ethanol-mediated oxidative tissue injury was evidenced by significantly down-regulated SOD, CAT, and a notable rise in the MDA contents in gastric tissue homogenates. Interestingly, METC pretreatment attenuated ethanol-oxidative stress in stomach tissues by strengthening gastric defense systems (increasing SOD and CAT production), which could be regarded as one of the main mechanisms of its gastroprotective potential. The antioxidant potential of METC is attributed to chemical contents, mainly its phenolic compounds (chlorogenic acid, quinic acid, and vitexin), which are consistent with previous studies highlighting their antioxidant properties [43,55]. In a similar study, *T. porrifolius* methanolic extract (50 and 250 mg/kg, groups II and III) exhibited a significant antioxidant potential, increasing SOD, GST, CAT, and reducing MDA generations in CCl4-induced hepatotoxic rats, which were mainly linked with its HPLC chemical profiles (quercetin, luteolin, gallic acid, and chlorogenic acid) [56]. Accordingly, *Tragopogon dubius* extracts attenuated lipopolysaccharide-induced ROS formation in mice, explained by their increased phenolic contents (coumaric acid, caffeic acid, and gallic acid) [57].

**Fig 10:**
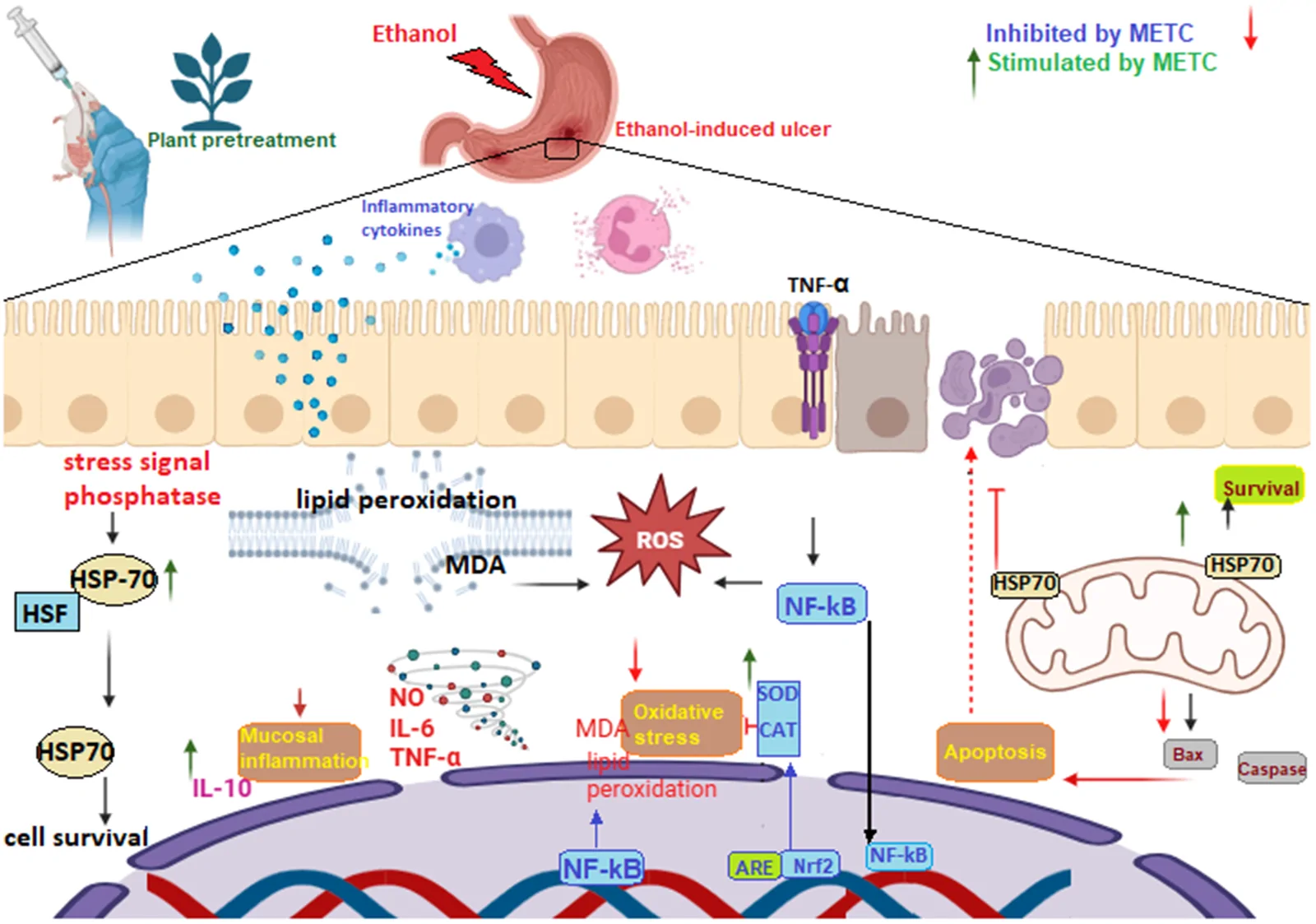
Shows the cellular mechanistic events attributed to the gastroprotective potentials of METC in the ulcerated rat model. A single intragastric ethanol delivery provoked ROS formation, lipid peroxidation, activated inflammatory-related pathways (Nrf2 and NF-κB), and intrinsic apoptotic pathways. METC pretreatment lowered oxidative stress and inflammatory response by increasing expression of endogenous antioxidants, anti-inflammatory cytokines, and anti-apoptotic proteins, altogether improved the gastric defense system, and lowered gastric mucosal injuries provoked by EtOH. The figure is created via bio render application (BioRender.com).

Inflammation is considered a crucial cellular process associated with ethanol-induced gastric ulcer, which is initiated by ROS generation and provoked macrophages producing numerous inflammatory cytokines IL-1β, IL-6, and TNF-α. The pro-inflammatory cytokines aggravate gastric injury by attracting neutrophils into inflammation site, causing connexin breakdown and destruction of mucosal barriers [58]. Moreover, ethanol can stimulate oxidative and inflammatory processes in gastric tissues by generating highly reactive byproducts like acetaldehyde and ROS molecules that severely drain endogenous antioxidant reserves, enhance mitochondrial dysfunction, and impair cellular structures. Concurrently, ethanol damages gastric barriers, enhancing endotoxins (LPS) leakage into the bloodstream, which hyper-activates immune cells, causing the release of pro-inflammatory cytokines, inflammation, and tissue injury [46]. The inflammatory chemicals can exacerbate the ROS generation in gastric tissues, enhancing further gastric ulcer formation. Our results were parallel with previous data [59], showing increased inflammation in gastric tissues as a result of intragastric ethanol delivery, denoted by up-regulated TNF-α and IL-6 cytokines and reduced IL-10 in tissues as compared to normal controls. While METC pretreatment exhibited significant anti-inflammatory potentials, evidenced by a significant reduction of TNF-α and IL-6 cytokines and a higher IL-10 level than ulcer controls. Such anti-inflammatory actions of METC could be linked with its phenolic contents, namely, coumaric acid, caffeic acid, and gallic acid, which are considered as effective antioxidants in different *in vivo* trials [15,60,61]. These outcomes concurs previous pathological results detailing reduced inflammatory response in different disease models treated with *Tragopogon* extracts [62,63]. Similarly, daily oral treatment of ethanol extract of *Tragopogon* (20, 30, or 50 mg/kg/d) reduced TNF-α and IL-1β, which are considered the main underlying pathways of healing potentials in TNBS-induced colitis in rats [44]. The possible gastroprotective mechanisms regulated by METC are hypothesized in Fig 10.

## Conclusion

The study investigates the acute toxicity and gastroprotective effects of METC in ethanol-induced ulcer in rats. The results non-toxic effects of METC in rats administered with up to 5 g/kg. The results suggested a noticeable gastroprotective potential of METC in the ethanol-induced ulcerogenesis model. METC improved gastric defense barriers by reducing mucus membrane permeability, increasing gastric pH, and increasing alcian blue binding capacity (**gastric wall mucus**/mucopolysaccharides) that ultimately protected the gastric mucosal layer from direct ethanol insults. The gastroprotective effects of METC have been attributed to its antioxidant (increased SOD, CAT, and decreased MDA) and anti-inflammatory (decreased TNF-α and increased IL-10) potentials that interrupted the oxidative stress-inflammation circle provoked by ethanol insults. Moreover, METC remarkably mimicked ethanol-mediated intrinsic/mitochondrial pathway of apoptosis in mucosal tissues via up-regulating HSP 70 expression and reducing Bax protein expression. This is the first preliminary evidence for the safe dosage and gastroprotective effect of METC against gastritis. The study limitations include a lack of detailed phytochemical analysis, the lack of direct molecular pathway analyses, and the use of a single acute ethanol-induced gastric ulcer model; therefore, future stronger molecular validation, phytochemical characterization (HPLC or LC-MS/MS), as well as chronic ulcer model along with depicted mechanistic events (NF-κB, PI3K/Akt, and Nrf2) are suggested to a better understand the anti-ulcer effects of METC.

## Abbreviation

ABB: Alcian blue binding
Bax: Bcl-2-associated X protein
CAT: catalase
H&E: hematoxylin and Eosin
HSP 70: heat shock protein
IL-6: interleukin-6
MDA: malondialdehyde
NF-κB: Nuclear Factor kappa-light-chain-enhancer of activated B cells
Nrf2: Nuclear factor erythroid 2-related factor 2
PAS: periodic-acid Schiff
PI3K/Akt: Phosphoinositide 3-kinase/Protein Kinase B
SOD: superoxide dismutase
TNF-α: tumor necrosis factor-α
